# P-1632. Living in an area with high burden outpatient antibiotic prescriptions, a risk factor for infection by ESBL producing Enterobacterales?

**DOI:** 10.1093/ofid/ofae631.1798

**Published:** 2025-01-29

**Authors:** Michael T Stevens, Patrick Kinsella, Darien Campbell, Rafael Ponce, Andrea Gonzalez

**Affiliations:** Mercer University School of Medicine, Macon, Georgia; Atrium Health Navicent The Medical Center, Macon, Georgia; Atrium Health Navicent, Macon, Georgia; Mercer University School of Medicine, Macon, Georgia; Atrium Health Navicent - Mercer University, Macon, Georgia

## Abstract

**Background:**

The increase in antibiotic resistance has been linked to antibiotic exposure. However, initiatives of antibiotic stewardship programs have been associated with decrease in antibiotic utilization but without a clear decrease in antibiotic resistance. We hypothesized that patients hospitalized with infections with ESBL producing Enterobacterales (ESBL-PE) live in areas with high outpatient antibiotic exposure.

Outpatient antibiotic prescriptions per zip code

**Figure:**
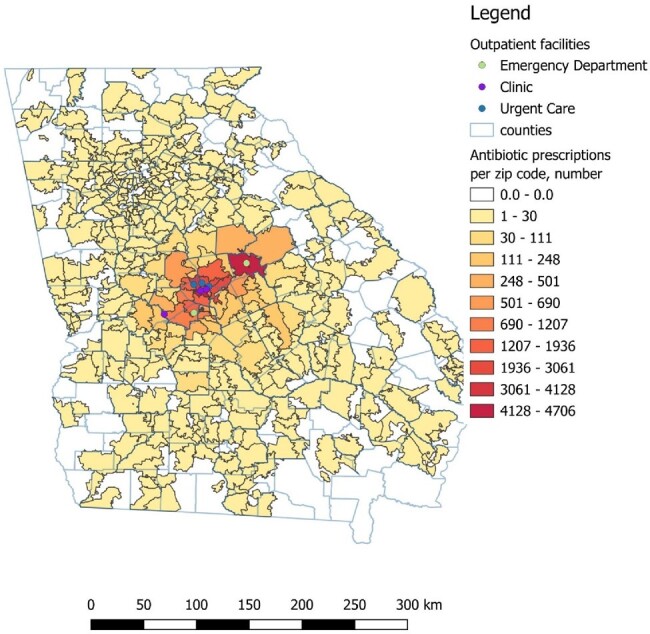

Antibiotics prescribed in the outpatient setting per zip code of residence of the recipient of the prescription.

**Methods:**

From March 2022 to March 2023 we evaluated antibiotic utilization in the outpatient setting of a healthcare system in central Georgia. Outpatient settings included urgent cares, emergency rooms, and primary care clinics. We estimated the antibiotic “burden” per zip code based on the residence of each patient receiving a prescription from these outpatient facilities.

During the same time period, we obtained all cultures processed in the hospital’s laboratory that isolated an ESBL-PE, and the zip code of residence from each patient’s sample.

Number of antibiotics per zip code was the independent variable and number of residents per zip code hospitalized with an ESBL-PE infection was the dependent variable. QGIS v3.8 was used for spatial analysis and SPSS 25 for statistical analysis.

Number of cases of an ESBL-PE infection per zip code of patient residence

**Figure:**
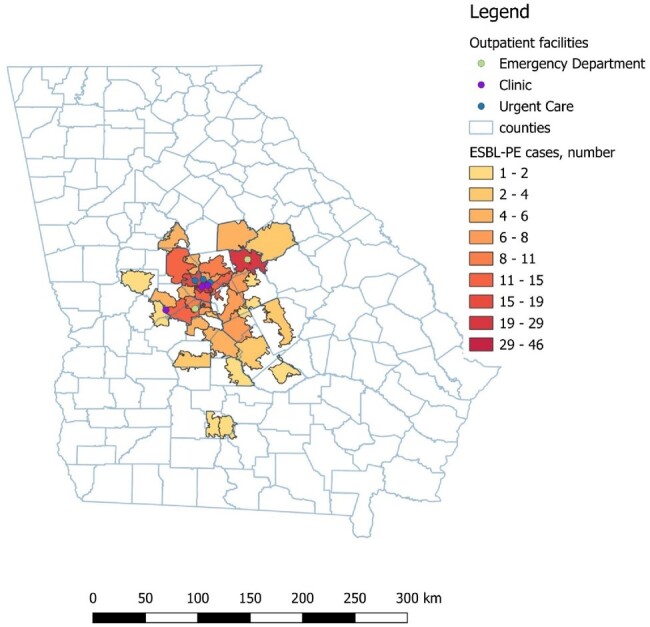

**Results:**

There were 61 070 outpatient prescriptions, representing 500 092 days of therapy. Prescriptions were given to residents of over 1000 different zip codes of nearly 20 states. 70% (42719) of these prescriptions were for residents of the state of Georgia, distributed among 634 zip codes. The zip code with the largest antibiotic burden received 4706 prescriptions in a one-year period, representing 161 antibiotic prescriptions per 1000 population.

396 Georgia residents had a culture with an ESBL-PE, and 308 (77.7%) of them were hospitalized for infections due to ESBL-PE. Linear regression model had an R2 of 0.79 with a p-value of < 0.01.

**Conclusion:**

The geographic reach of antibiotic prescriptions by outpatient facilities is of concern. The antibiotic burden in surrounding communities is higher than areas further away. Living in an area with high outpatient antibiotic prescription burden explained almost 80% of the variance for hospitalization with an infection due to ESBL-PE. Efforts to decrease antibiotic resistance should also include interventions in outpatient facilities.

**Disclosures:**

**All Authors**: No reported disclosures

